# Comparison of cardiorespiratory and anesthetic effects of ketamine-midazolam-xylazine-sufentanil and tiletamine-zolazepam-xylazine in miniature pigs

**DOI:** 10.1371/journal.pone.0271325

**Published:** 2022-07-12

**Authors:** Zhiheng Zhang, Hui Bai, Bo Zhang, Meilun Shen, Li Gao

**Affiliations:** 1 College of Veterinary Medicine, Northeast Agricultural University, Harbin, Heilongjiang, China; 2 Heilongjiang Key Laboratory for Laboratory Animals and Comparative Medicine, College of Veterinary Medicine, Northeast Agriculture University, Harbin, China; Cardiac Surgery Unit, Papardo Hospital, ITALY

## Abstract

Effective and adequate anesthesia is conducive to better restrain for pigs during surgical or diagnostic procedures This study aimed to evaluate cardiorespiratory and anesthetic effects of ketamine-midazolam-xylazine-sufentanil or tiletamine-zolazepam-xylazine as general anesthetics in miniature pigs. In phase 1, one of the combinations was administered intramuscularly to miniature pigs. The KMXS protocol combined 10 mg kg^-1^ ketamine, 0.5 mg kg^-1^ midazolam, and 2 mg kg^-1^ xylazine with 2 μg kg^-1^ sufentanil. The TZX protocol combined 2.2 mg kg^-1^ tiletamine, 2.2 mg kg^-1^ zolazepam, and 1.4 mg kg^-1^ xylazine. After treatment, the mean arterial pressure, systolic arterial pressure, diastolic arterial pressure, heart rate, respiratory rate, peripheral hemoglobin oxygen saturation, rectal temperature, and anesthesia quality were recorded. In phase 2, the feasibility of KMXS and TZX as general anesthetics were evaluated for pig castration. In phase 1, both drug combinations provided smooth induction with similar anesthetic effects. The KMXS protocol provided moderate anesthesia for 60–70 minutes in pigs, while the TZX protocol provided 30–45 minutes. In phase 2, castration was completed smoothly in all pigs with little fluctuation in physiological variables. The KMXS protocol is better for medium-term anesthesia, while the TZX protocol is preferable for short-term anesthesia in pigs.

## Introduction

Pigs are frequently used animal models for medical research because pig cardiorespiratory anatomy and physiology are similar to humans [1]. Pigs are difficult to restrain and anesthetize effectively, yet adequate anaesthesia of pigs is often required in clinical veterinary practice for surgical or diagnostic procedures [2–4]. Intramuscular (IM) anesthesia is preferred for pigs that are difficult to anesthetize intravenously [5]. However, no single anesthetic agent is adequate for pigs during surgical operations. Thus, two or more drug combinations are needed [6–9]. The intramuscular drugs include dissociative agents (tiletamine and ketamine), benzodiazepines (zolazepam and midazolam), and α2-adrenergic receptor agonists (xylazine and dexmedetomidine) [6, 9, 10].

Tiletamine is a competitive N-methyl-D-aspartic acid (NMDA) receptor antagonist that exerts analgesia and immobilization, while zolazepam is a benzodiazepine drug for muscle relaxation and sedation [11–13]. Xylazine is an α2 receptor agonist for sedation, analgesia, and muscle relaxation [14]. The combination of tiletamine, zolazepam, and xylazine (TZX), is widely used to immobilize and effectively anesthetize pigs in veterinary practices [7]. However, the TZX combination is associated with adverse effects during the recovery, including ataxia and uncontrolled movements [7].

Therefore, ketamine, a non-competitive NMDA receptor antagonist [15] and midazolam, a benzodiazepine sedative [16] are commonly used in various animals. The combination of ketamine (20 mg kg-1)-midazolam(0.25 mg kg-1)-xylazine (2 mg kg-1) provide sufficient anesthesia for pigs within a short time but with an adequately long duration of anesthesia [17].

Nonetheless, opioids are another widely used analgesic. Sufentanil is a μ-opioid agonist with a 5–10 times higher analgesic effect than its parent drug, fentanyl. Thus, sufentanil is clinically used in general anesthesia and analgesia for dogs [18–21].

However, there are no published manuscripts of veterinary medicine on the use of ketamine, midazolam, xylazine plus sufentanil (KMXS) drug combination for pig anesthesia. We hypothesized that KMXS provides adequate medium-term general anesthesia for pigs with few side effects that could be used for surgical procedures in clinical veterinary practice. Thus, this study evaluated the KMXS combination to provide knowledge on KMXS used for pig anesthesia. The study was performed in two phases to determine the KMXS efficacy in pigs using physiological parameters and evaluating the level of anesthesia (phase 1). Phase 2 evaluated the feasibility of combining KMXS as a general anesthetic for pig castration. All the results of the KMXS treatment were compared with the corresponding effects of TZX.

## Materials and methods

### Animals

The Laboratory Animal Welfare and Ethics Committee of Northeast Agricultural University (Harbin, China, #NEAU-2016-06-1324-29) approved the study, performed following the guidelines for Animal Experiments at the Northeast Agricultural University. The study included 36 healthy, eight-month-old Chinese experimental miniature pigs (28 males and 8 females) weighing 36.2 ± 4.8 kg. Pigs were ear tagged for proper identification. The pigs were placed in individual rooms maintained at 23±0.5°C temperature and 55%±15% humidity in an animal research facility at the Northeast Agricultural University of Life Sciences. The room lights were on/off at 7:00 am/ 9:00 pm, and individual room windows provided additional natural light. Pigs were provided a combination of a commercial pig diet and hay with water ad libitum. A physical examination and complete blood count revealed that all the miniature pigs were healthy. Phase 1 included 16 pigs (half male and half female), while phase 2 had 20 male pigs.

### Phase 1: Comparison of cardiopulmonary and anesthetic effects of KMXS and TZX

#### Study design

Sixteen male pigs were allocated into two equal groups (n = 8, half male and half female), using a random number table, namely KMXS group (ketamine-midazolam-xylazine-sufentanil) or TZX group (tiletamine-zolazepam-xylazine). The pigs fasted overnight with ad libitum access to water. All the investigators involved in both experiments were unaware of the treatment administered to individual animals.

#### Anesthesia protocol

Food, but not water, was withheld for 12 hours before drug treatments, and all pigs were acclimatized for at least 30 minutes in a room set at 25°C. All pigs are considered healthy following clinical examination and complete blood count before each anesthesia, and each animal was weighed on the day of the experiment. Anesthesia was induced with either KMXS (a combination of ketamine 10 mg kg^-1^, midazolam 0.5 mg kg^-1^, xylazine 2 mg kg^-1^, and sufentanil 2 μg kg^-1^), or TZX (a combination of 2.2 mg kg^-1^ tiletamine, 2.2 mg kg^-1^ zolazepam, and 1.4 mg kg^-1^ xylazine), administered IM in the neck muscles to the base of the ear. Each combination of drugs was mixed in the same syringe immediately before injection. The recumbent pigs were positioned in the right lateral recumbency and allowed to breathe freely.

#### Physiological values monitoring

Physiological values, including heart rate (HR), respiratory rate (RR), mean arterial pressure (MAP), systolic arterial pressure (SAP), diastolic arterial pressure (DAP), rectal temperature (RT), and peripheral hemoglobin oxygen saturation (SpO_2_) were recorded before treatment (baseline), and at 0, 5, 10, 20, 30, 45, 60, 75 and 90 minutes after treatment. The HR, noninvasive blood pressure (NIBP, including MAP, SAP, and DAP, oscillometry), SpO_2_ and RT, were monitored with the iMCE8 Vet physiological multi-parameters monitor (Mindray, Guangdong, China). However, the HR, SPO_2_, and RT were measured using electrocardiograph (ECG) clips, SpO_2_ tongue, and temperature probes. For each pig, the NIBP was measured by oscillometry using a blood pressure cuff on the right thoracic limb, slightly distal to the elbow. The RR was recorded by observing thoracic movements for one minute or physiological multi-parameters. The baseline HR was measured manually using a stethoscope placed at the lower-left lateral thoracic wall. All physiological values were recorded by the same investigator who monitored each pig until after recovery.

#### Quality assessment of anesthesia

Three evaluators blinded to the experimental details independently assessed anesthesia quality, including induction time, anesthesia duration, duration before the onset of sternal recumbency, standing and walking, and mean anesthesia scores. Induction time is the time from injection to complete recumbency. Moreover, the anesthesia duration refers to the time interval between complete recumbency and the first attempt made by the animal to lift its head a few centimeters above the ground. The duration before the onset of sternal recumbency refers to the time interval between the end of anesthesia until the animal started sternal recumbency. The time to standing refers to the time interval between the end of anesthesia until the animal was standing without assistance for longer than 10 seconds. The time to walking refers to the time from standing until when the animal could walk a short distance [22, 23].

Anesthesia scores were measured using previously reported methods [24–26] at 5, 10, 20, 30, 45, 60, 75, and 90 minutes after treatment. The total score was the sum of posture, sedation, analgesia, skeletal muscle relaxation, and auditory response score according to the criteria described in Table 1 [25, 27]. The analgesia score was assessed by reflex withdrawal to clamping of the tail, the skin of body surface at the paramedian abdomen, and the interdigital foot web of all limbs for 3 seconds using Kocher’s forceps [27]. The auditory response is defined as the response to the noise created by a handclap close to the animal’s ears. the greater the score, the better the quality of anesthesia. A maximum score of 15 indicated excellent anesthesia, while scores of 11–14 indicated moderate anesthesia, and a score <11 indicated mild anesthesia [25, 26].

**Table 1:** Anesthesia score.

Criteria	Score	Observation
Posture score	0	Standing
	1	Sitting
	2	Sternal recumbency
	3	Lateral recumbency
Sedation score	0	Normal
	1	Mild sedation (recumbent, head down, strong palpebral reflex, normal eye position)
	2	Moderate sedation (recumbent, head down, moderate palpebral reflex, partial ventromedial eye rotation)
	3	Profound sedation (recumbent, head down, palpebral reflex absent, complete ventromedial eye rotation)
Analgesia score *	0	Normal response (productive flight response)
	1	Mild (exaggerated movements of limbs and trying to get up)
	2	Moderate (slight movements of the limbs and trying to get up)
	3	Profound (lack of response)
Muscle relaxation score	0	Normal jaw and leg tone
	1	Mild relaxation of jaw and leg tone
	2	Moderate relaxation and leg tone
	3	Profound relaxation of jaw and leg tone
Auditory response †	0	Normal response
	1	Mild decrease in response (eye movement with body movement)
	2	Moderate decrease in response (eye movement without body movement)
	3	Profound decrease in response (no movement)

*Reflex withdrawal to clamping of the tail, the skin of body surface at the paramedian abdomen, and the interdigital web of the foot of all limbs for 3 seconds using Kocher’s forceps.

†Response to noise created by a handclap close to the animal’s ears

#### Adverse events

The occurrence of adverse events was recorded, including myoclonus, regurgitation, cardiac arrest, hypoventilation (f_R_ < 6 breaths minute-1), hypoxemia (SpO_2_ < 90%), bradycardia (HR < 60 beats minute-1), hypotension (MAP < 60 mmHg), or hypothermia (T < 36.6°C). Any pig showing adverse events during the experiment was excluded from the study and treated appropriately.

### Phase 2: KMXS and TZX use in a surgical procedure

#### Preoperative preparation and the surgical procedure

The phase 2 surgical castration involved 20 male pigs, which were allocated into two equal groups (n = 10), using a random number table. The pigs fasted overnight with ad libitum access to water. The KMXS or TZX anesthesia protocol was administered by IM injection into the neck muscles (ten pigs per group). Once anesthetized, each pig was positioned in the dorsal recumbency to prepare for surgery using 100% oxygen supplied by the mask. The caudal ventral abdomen and/or perineum was clipped, aseptically prepared, and 2% lidocaine was injected into the testes. All pigs were castrated following the pre-scrotal approach [28], in which pressure is applied on the scrotum to position the testicles to the pre-scrotal area. The skin and subcutaneous tissues were incised along the median raphe or parallel to the median raphe over the displaced testicle. The testicle was exteriorized by manual dissection of the surrounding soft tissues. The pampiniform plexus and vas deferens were ligated by a transfixion ligature of 2–0 polyglycolic acid suture. The surgical incisions were stretched manually and left open. The same veterinary student performed all surgical procedures. Amoxicillin-clavulanate potassium and meloxicam were administered after surgery.

#### Measurement of parameters

The HR, RR, MAP, SAP, DAP, T, SpO_2_, and anesthesia scores were recorded 0, 5, 10, 20, 30, 45, 60, 75, and 90 minutes after administration of KMXS or TZX. The start time of surgery, duration of surgery (minutes) from incision to completion, and the time from completion of surgery until the pig was standing, was recorded. Adverse reactions (myoclonus, regurgitation, cardiac arrest, hypoventilation, hypoxemia, bradycardia, hypotension, or hypothermia) were recorded during the surgery.

### Statistical analysis

The sample size was estimated from preliminary data with 80% power to detect a 15% difference in anesthesia score between different treatment groups. The alpha was set at 5%; thus, a minimum of eight pigs was required per group.

The Shapiro-Wilk test determined the normality of data distribution. Data are presented as means ± standard deviation for parametric variables and medians (min-max) for nonparametric variables. The student’s t-test assessed differences between normally distributed groups for induction time, anesthesia duration, recovery time, the time to onset of sternal recumbency, and standing and walking in phase 1. The t-test also assessed the start time of surgery, duration of surgery from incision to completion, and the time from completion of surgery until the pig was standing in phase 2. The general linear mixed model analyzed the normally distributed physiological values (HR, MAP, SAP, DAP, RR, SpO_2_, and RT). Time, treatment, and their interactions were fixed effects, and individual pigs were random effects. Post-hoc multiple comparisons were performed using student’s t-test to compare treatment groups at the same time points. Anesthesia scores were not normally distributed (nonparametric variables). Thus, a generalized linear mixed-effects model assuming a Poisson family with a logarithmic link function was used. Simultaneously, Mann-Whitney U tests compared scores at different treatment groups and the 5-minutes time point. Values with p < 0.05 were considered significantly different.

## Results

### Anesthesia quality in phase 1

All the pigs were anesthetized with two drugs combination in phase 1. Lateral recumbency was induced rapidly within four minutes in both groups of pigs after drug treatments. The induction time, recovery, sternal recumbency, standing, and walking were not significantly different between groups (Table 2). The KMXS duration time was significantly longer than the TZX group (p < 0.001). All pigs in the KMXS group smoothly recovered without ataxia and emergence delirium. The pigs stood and walked after one or two attempts. However, ataxia, uncontrolled movements, and emergence delirium were observed in six pigs recovering from TZX.

**Table 2:** The times of induction, duration, the times to onset of sternal recumbency, standing and walking in pigs that were treatment with ketamine-midazolam-xylazine-sufentanil (group KMXS) or tiletamine-zolazepam-xylazine (group TZX) in phase 1.

Variables	KMXS	TZX	*p* -value
Time of induction (minutes)	2.9±0.8	3.8±1.3	0.089
Time of duration (minutes)	76.1±8.6	54.1±10.4*	< 0.001
Sternal recumbency	13.5±4.6	11.2±2.9	0.243
Standing	11.6±4.3	12.3±5.1	0.794
Walking	6.4±3.0	3.9±2.1	0.082

Values are expressed as mean ± standard deviation (SD).

* Significantly difference between treatments groups (*p* < 0.05).

**Fig 1:**
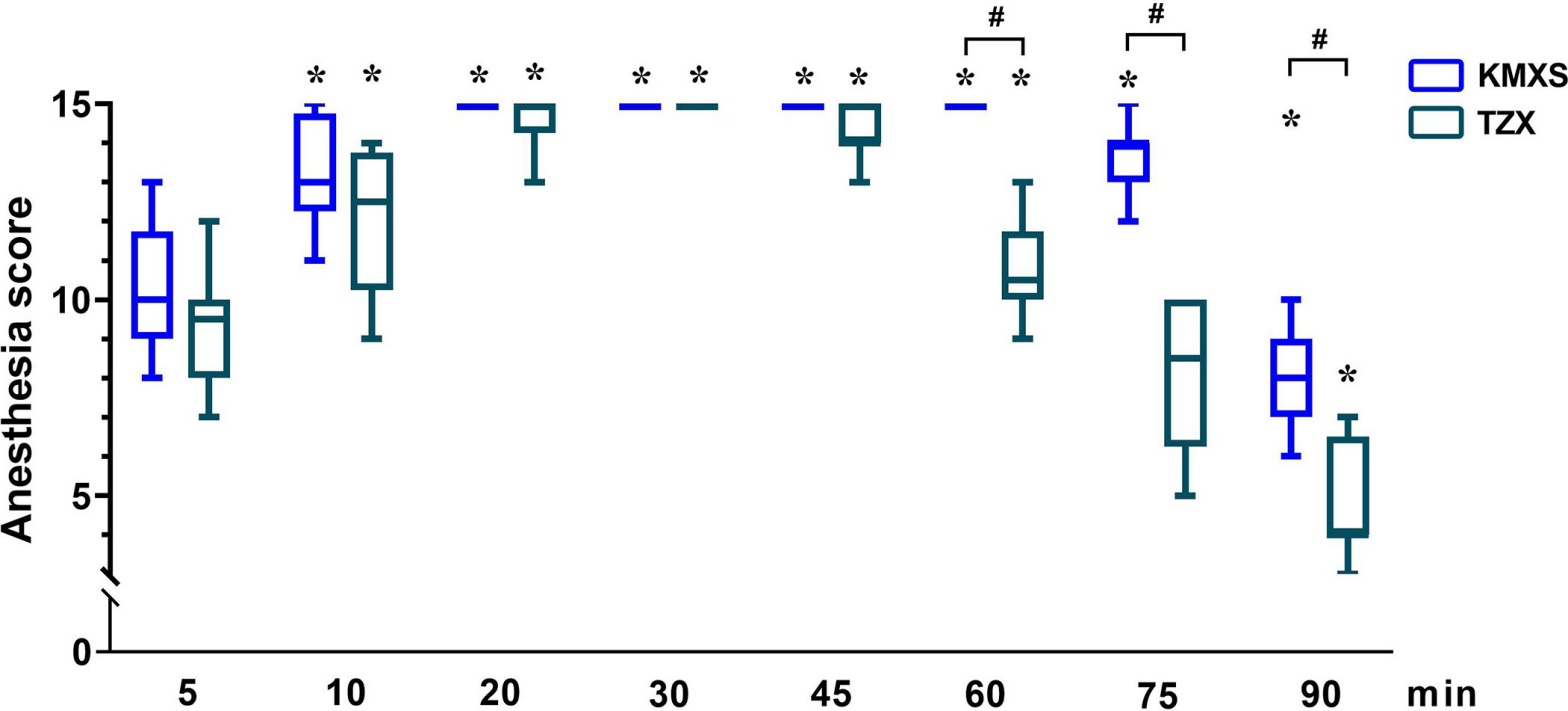
Anesthesia scores in pigs (n = 8) undergoing anesthesia with KMXS or TZX protocol at phase 1. KMXS protocol, 10 mg kg^-1^ ketamine, 0.5 mg kg^-1^ midazolam, and 2 mg kg^-1^ xylazine, plus 2 μg kg^-1^ sufentanil. TZX protocol 4.4 mg kg^-1^ tiletamine-zolazepam plus 1.4 mg kg^-1^ xylazine. Values are expressed as medians (min-max). * Significantly different from baseline at the same group (*p* < 0.05); ^#^ Significantly different between treatment groups (*p* < 0.05).

Both KMXS and TZX produced satisfactory general anesthesia in all pigs. The anesthesia score, shown in Fig 1, was statistically significant over time (p < 0.001). The interaction between time and treatment was significantly different (p = 0.001). The anesthesia score was significantly lower in the TZX than in the KMXS groups (p < 0.001). Overall, the anesthesia scores first increased and later decreased in both KMXS and TZX groups. The interaction between time and treatment was higher in the KMXS than in the TZX group (p < 0.001). The mean time for anesthesia scores > 11 (in minutes, with 95% confidence interval) was 66 (95% CI = 59 to 73) in KMXS, and 40 (95% CI = 30 to 49) in TZX. The mean time for analgesia scores at 3 points (in minutes, with 95% confidence interval) was 57 (95% CI = 51 to 62) in KMXS and 38 (95% CI = 31 to 43) in TZX.

### Physiological values in phase 1

**Fig 2:**
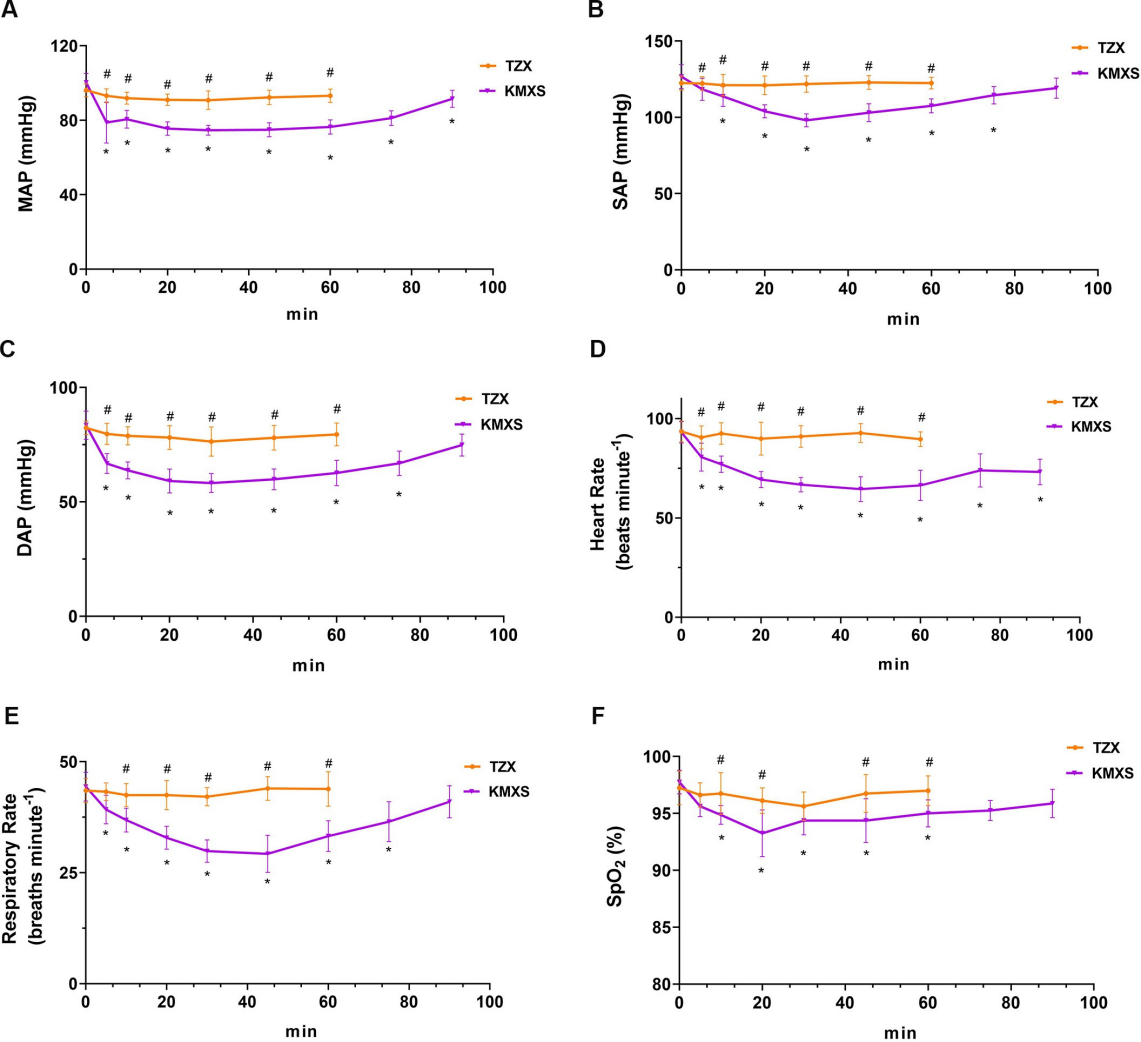
Cardiopulmonary variables in pigs (n = 8) that were administered with with KMXS or TZX protocol at phase 1. (A) mean arterial pressure (MAP), (B) systolic arterial pressure (SAP), (C) diastolic arterial pressure (DAP), (D) Heart rate (HR), (E) Respiratory rate (RR), and (F) peripheral hemoglobin oxygen concentration (SpO_2_). KMXS protocol, 10 mg kg^-1^ ketamine, 0.5 mg kg^-1^ midazolam, and 2 mg kg^-1^ xylazine, plus 2 μg kg^-1^ sufentanil. TZX protocol (n = 8), 4.4 mg kg^-1^ tiletamine-zolazepam plus 1.4 mg kg^-1^ xylazine. Values are expressed as mean ± standard deviation (SD). *Significantly different from baseline at the same group (*p* < 0.05); ^#^ Significantly different between treatment groups (*p* < 0.05).

The results of physiological parameters are shown in Fig 2. Eight pigs in group TZX gradually awakened after 60 minutes, and the subsequent physiological parameter data were not recorded. Still, in TZX, the MAP, SAP, and DAP changed negligibly, but the NIBP in the KMXS group decreased between 5 and 20 minutes and later normalized. The MAP, SAP, and DAP were significantly higher in TZX than KMXS (p < 0.001). Similarly, SAP, DAP, and MAP varied negligibly between the time and treatment groups. The SAP, DAP, and MAP were not significantly different over time.

In the KMXS group, HR decreased within 5 minutes and remained consistently below baseline for 90 minutes, and the declining rate from baseline was 10~25 beats per minute. However, the HR in the TZX group was not statistically significant compared with the baseline. Furthermore, the HR varied significantly in the treatment group, time, and their interactions (p = 0.001, 0.005, and 0.049, respectively).

The SpO2 and RR were not statistically different over the time course and the interaction between time and treatment groups. Nevertheless, SpO_2_ and RR were significantly higher in the TZX than in the KMXS group (p = 0.036 and 0.001), but the baseline RR and SPO_2_ were not significantly different from TZX. In the KMXS group, both SpO_2_ and RR were consistently below the baseline at 5~ 90 minutes, and some pigs developed mild hypoxemia. The RT decreased relative to the baseline after KMXS or TZX treatments but was non-significant. The rectal temperature of all pigs was 38.0 ± 0.5°C. No hypotension, bradycardia, or bradypnea was observed in phase 1, except for mild hypoxemia in the KXMS group.

### Anesthesia quality in phase 2

**Fig 3:**
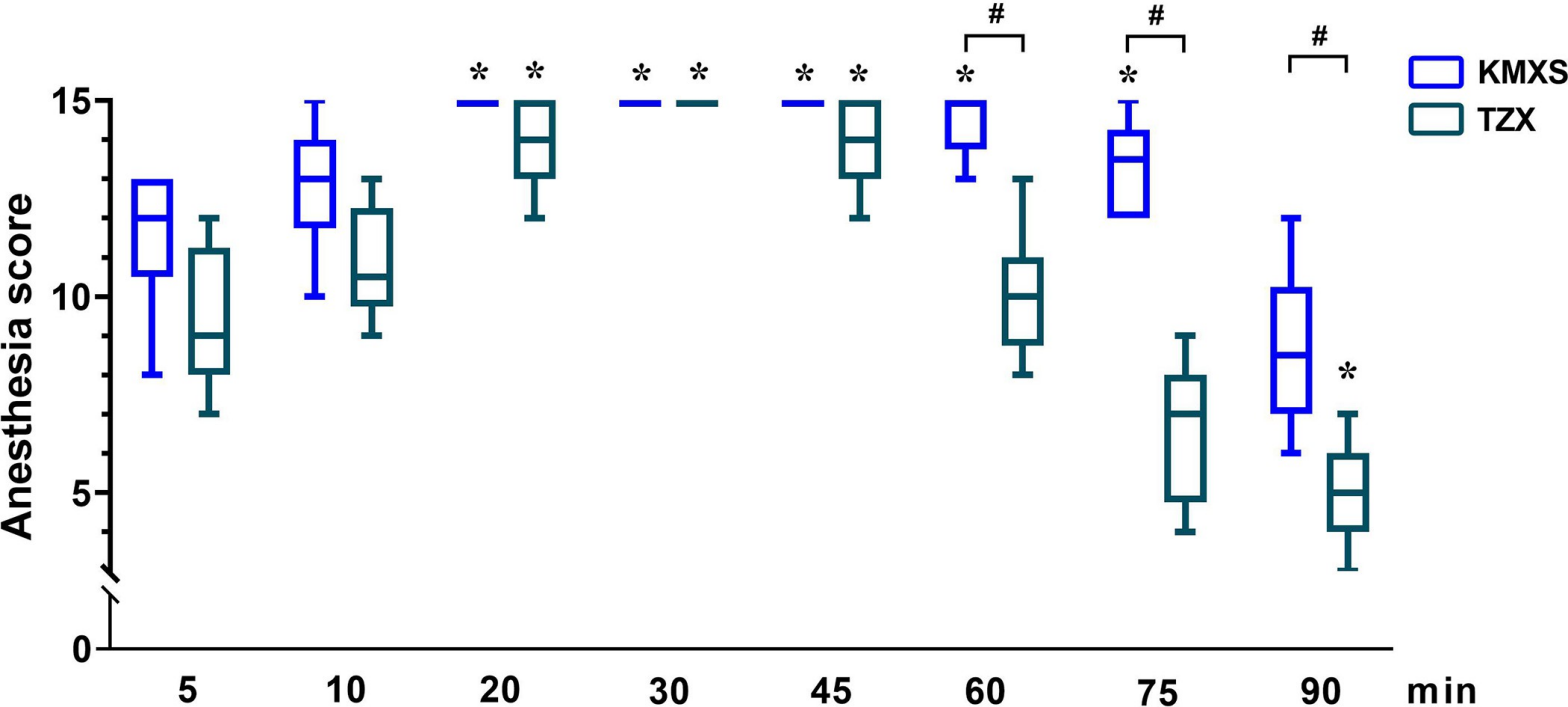
Anesthesia scores in pigs (n = 10) undergoing castration with KMXS or TZX protocol at phase 2. KMXS protocol, 10 mg kg^-1^ ketamine, 0.5 mg kg^-1^ midazolam, and 2 mg kg^-1^ xylazine, plus 2 μg kg^-1^ sufentanil. TZX protocol, 4.4 mg kg^-1^ tiletamine-zolazepam plus 1.4 mg kg^-1^ xylazine. Values are expressed as medians (min-max). * Significantly different from baseline at the same group (*p* < 0.05); ^#^ Significantly different between treatment groups (*p* < 0.05).

In phase 2, all twenty pigs underwent castration smoothly with no apparent movement or pain during surgery. The anesthesia score (Fig 3) was significantly lower in the TZX than in the KMXS group (p < 0.001). The overall trend of anesthesia scores first increased at 5~20 minutes then decreased at 60~90 minutes in both KMXS and TZX groups. Considering the interaction between time and treatment, TZX was significantly lower than KMXS (p < 0.001). The mean time when the anesthesia score was over 11 was 55 (95% CI = 48 to 62) in KMXS and 34 (95% CI = 29 to 42) in TZX. The mean time for analgesia score at 3 points was 56 (95% CI = 49 to 60) in KMXS and 34 (95% CI = 28 to 39) in TZX. Time related to surgery is summarized in Table 3. The surgery completion time until the pig was standing in KMXS was significantly longer than TZX (p < 0.001).

**Table 3:** Time related with surgical procedures at phase 2.

Variables	KMXS	TZX	*p* -value
Surgery start time	18.5±2.9	19.9±3.7	0.366
surgery duration (from incision to completion)	12.8±3.2	13.7±2.5	0.488
the time from completion of surgery until the pig standing	46.7±7.9*	28.5±5.9	< 0.001

Values are expressed as mean ± standard deviation (SD

* Significantly difference between treatments groups (*p* < 0.05).

### Physiological values in phase 2

**Fig 4:**
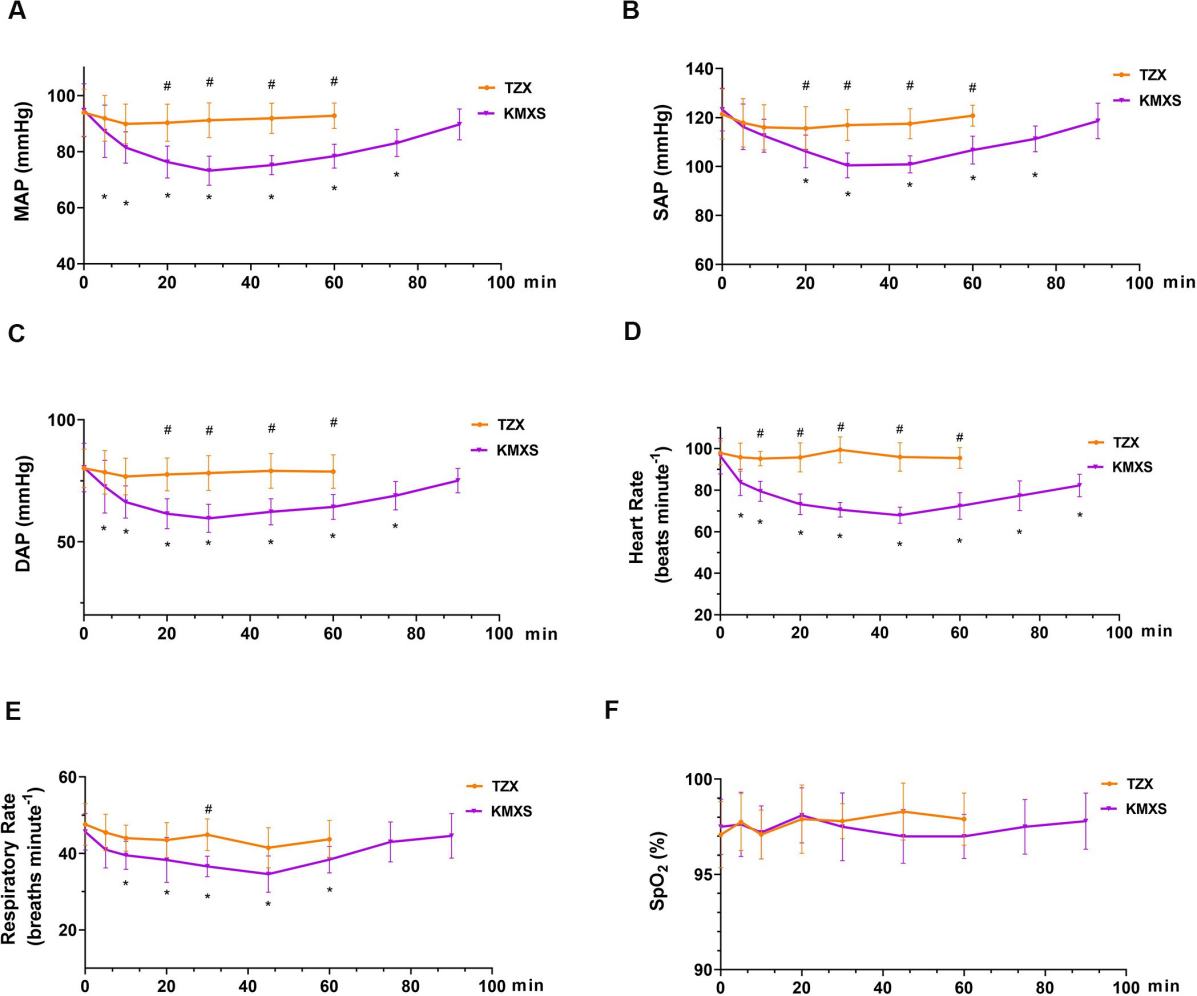
Cardiopulmonary variables in pigs (n = 10) that were treatmented with with KMXS or TZX protocol at phase 1. (A) mean arterial pressure (MAP), (B) systolic arterial pressure (SAP), (C) diastolic arterial pressure (DAP), (D) Heart rate (HR), (E) Respiratory rate (RR), and (F) peripheral hemoglobin oxygen concentration (SpO_2_). KMXS protocol, 10 mg kg^-1^ ketamine, 0.5 mg kg^-1^ midazolam, and 2 mg kg^-1^ xylazine, plus 2 μg kg^-1^ sufentanil. TZX protocol (n = 8), 4.4 mg kg^-1^ tiletamine-zolazepam plus 1.4 mg kg^-1^ xylazine. Values are expressed as mean ± standard deviation (SD). *Significantly different from baseline at the same group (*p* < 0.05); ^#^ Significantly different between treatment groups (*p* < 0.05).

The results of physiological parameters in phase 2 are shown in Fig 4. The physiological parameters were not recorded after the animals recovered. In TZX, the MAP, SAP, and DAP changed negligibly, while the MAP, SAP, and DAP in the KMXS group decreased and then returned to baseline after treatment. The MAP, SAP, and DAP were significantly higher in TZX than KMXS (p < 0.001). The MAP, SAP, and DAP variations were non significantly over time course or among interaction between time and treatment groups (p > 0.05). In the KMXS group, HR decreased within 5 minutes and remained consistently below baseline for 90 minutes. The mean HR declined from the baseline by 10~20 beats per minute, while the HR in TZX was not statistically different from the baseline (p > 0.05). During phase 2, SpO2 was not statistically different over time, treatment groups, and their interaction, and all values were within the normal range. RR was statistically different over time, treatment groups, and their interaction (p = 0.035, 0.001, and 0.040, respectively), but all values were within the normal range. The RT of all pigs was 38.2 ± 0.5°C. No hypoxemia, hypotension, bradycardia, bradypnea, or complications associated with surgery were observed during phase 2.

## Discussion

In this study, cardiorespiratory and anesthetic effects were evaluated after IM administration of KMXS (phase 1), and the feasibility of KMXS as a general anesthetic in pig castration was subsequently evaluated (phase 2). The KMXS combination had not previously been tested in pigs, and all the results were compared with TZX. In phase 1, KMXS caused rapid and smooth induction in anesthetized pigs. The TZX group also reported rapid and smooth anesthetic induction with some adverse events, consistent with previous studies [7, 9].

In another study, the anesthesia effects of 20 mg kg^-1^ ketamine (K20MX) or 10 mg kg^-1^ ketamine (K_10_MX) in combination with midazolam (0.25 mg kg^-1^) and xylazine (2 mg kg^-1^) was compared in pigs [17]. K_10_XM lacked analgesic effects with shorter recumbency and recovery duration. The K20XM group can provide analgesia within 40 minutes, but the duration of anesthesia is significantly longer (approximately 90 minutes) [17]. We hypothesize that the addition of opioids is necessary to improve the K10XM effectiveness. Based on the preliminary experiments from this study, the dose was optimized and adjusted to 10 mg kg^-1^ ketamine, 0.5 mg kg^-1^ midazolam and 2 mg kg^-1^ xylazine, and 2 μg kg^-1^ sufentanil.

In phase 1, The anesthesia score increased gradually (5~20 minutes) and then reduced (60~90 minutes) over time in both KMXS and TZX, but the KMXS score significantly increased compared with TZX over the time course, treatment, and their interaction. Moderate anesthesia was defined as the total anesthesia score > 11 [25, 26], and the duration of analgesia was defined as the time when the analgesia score was 3. The results indicate that KMXS provided 60~70 minutes of moderate anesthesia (duration of analgesia: 50~60 minutes) to pigs, while TZX provided 30~45 minutes (duration of analgesia: 30~40 minutes). Besides, we found that sufentanil addition improved the quality and duration of anesthesia (or analgesia) of KMXS, which was significantly higher than TZX.

Xylazine is associated with bradycardia, respiratory depression, and hypotension [29, 30], probably due to the decreased sympathetic nerve activity and/or increased vagus tone [31]. In this study, the TZX group had no bradycardia, hypotension, and respiratory depression. The arterial blood pressure did not change considerably in the TZX group but decreased (5~60 minutes) and returned to the baseline (90 minutes) in the KMXS group. The overall MAP, SAP, and DAP in the TZX was higher than KMXS without hypotension (MAP<60 mmHg). The decrease in arterial blood pressure (MAP, SAP, and DAP) was not clinically significant in the KMXS group and required no treatment. The HR in the KMXS group decreased within 5 minutes and remained consistently below the baseline, and the average HR decreased by 10~25 times per minute, while the HR in TZX did not change significantly. The mean arterial pressure decreased without an initial increase from previous reports, calves and pigs that received an intramuscular injection of xylazine. Still, the HR and cardiac output decreased, similar to the KMXS results in this study [9, 31, 32]. In addition, we hypothesize that different concentrations of xylazine (KMXS group 2 mg kg^-1^ vs TZX group 1.4mg kg^-1^) caused the difference in arterial blood pressure and heart rate between the two treatment groups in stage one.

Nevertheless, the RR and SpO_2_ were higher in the TZX than in the KMXS group. The KMXS group showed mild hypoxemia and respiratory depression at phase 1. The RT in the two treatment groups was not significantly different and remained within the normal range. Therefore, we believe that the KMXS protocol has a relatively small effect on the physiological variables of pigs, except for mild hypoxemia, which may require additional oxygen supplementation and monitoring.

Castration is one of the most commonly surgical procedures in domestic and pet pigs [28, 33, 34]. In phase 2, the feasibility of KMXS protocol as general anesthetics was evaluated in pigs through surgical castration. To improve the mild hypoxemia and respiratory depression of the KMXS protocol in phase 1, we provided additional oxygen to all pigs in phase 2 through a mask. As expected, the phase 2 results revealed that KMXS or TZX protocol completed the operation without complications. Meanwhile, our results indicated that the KMXS group provided the pigs with 50~60 minutes of moderate anesthesia (duration of analgesia: 50~60 minutes).

In contrast, the TZX group provided 30~40 minutes (duration of analgesia: 30~40 minutes) in phase 2. The MAP, SAP, DAP, and HR in the TZX group were higher than the KMXS group, and the changes were within the acceptable range. Similarly, all values of SpO2 and RR in the KMXS protocol were within the normal range. No hypoxemia and respiratory depression were observed, possibly due to additional supplemental oxygen.

This study had some limitations. First, the study monitored the non-invasive blood pressure, but the more reliable method of invasive blood pressure or doppler blood pressure was not monitored due to lack of equipment. Secondly, the study sample size was small. Future studies will evaluate more clinical applications on the KMXS protocol.

## Conclusions

In conclusion, these results support the combination of 10 mg kg^-1^ ketamine, 0.5 mg kg^-1^ midazolam, and 2 mg kg^-1^ xylazine with 2 μg kg^-1^ sufentanil administered intramuscularly for medium-term anesthesia. Tiletamine-zolazepam-xylazine is preferred for short-term anesthesia on pigs in clinical veterinary practice.

## Supporting information

S1 Data (XLSX)
